# Real-Time Analysis of a Sensor’s Data for Automated Decision Making in an IoT-Based Smart Home

**DOI:** 10.3390/s18061711

**Published:** 2018-05-25

**Authors:** Nida Saddaf Khan, Sayeed Ghani, Sajjad Haider

**Affiliations:** 1 Telecommunication Research Lab, Department of Computer Science, Institute of Business Administration, Garden/Kayani Shaheed Road, Karachi 74400, Pakistan; sghani@iba.edu.pk; 2 Artificial Intelligence Lab, Department of Computer Science, Institute of Business Administration, Garden/Kayani Shaheed Road, Karachi 74400, Pakistan; sahaider@iba.edu.pk

**Keywords:** sensor analytics, flowmeter, internet of things (IoT), real-time data, Artificial Neural Network (ANN), MSA forecasting

## Abstract

IoT devices frequently generate large volumes of streaming data and in order to take advantage of this data, their temporal patterns must be learned and identified. Streaming data analysis has become popular after being successfully used in many applications including forecasting electricity load, stock market prices, weather conditions, etc. Artificial Neural Networks (ANNs) have been successfully utilized in understanding the embedded interesting patterns/behaviors in the data and forecasting the future values based on it. One such pattern is modelled and learned in the present study to identify the occurrence of a specific pattern in a Water Management System (WMS). This prediction aids in making an automatic decision support system, to switch OFF a hydraulic suction pump at the appropriate time. Three types of ANN, namely Multi-Input Multi-Output (MIMO), Multi-Input Single-Output (MISO), and Recurrent Neural Network (RNN) have been compared, for multi-step-ahead forecasting, on a sensor’s streaming data. Experiments have shown that RNN has the best performance among three models and based on its prediction, a system can be implemented to make the best decision with 86% accuracy.

## 1. Introduction

The internet of thing (IoT) refers to sensing and collecting data from different devices about everyday physical phenomena [1]. IoT has gained wide popularity due to its huge market in various sectors such as healthcare, security, home automation, etc. To make an IoT system smart and artificially intelligent, data serves as a fuel which is generated by various sensors and devices within the system. Various data analytic techniques could be applied to these data to form strategic courses of action. One of the most widely used analytical techniques is time series analysis, which is used to understand past observations to learn an appropriate model. These models can be used not only to predict the future values, but also to learn significant pattern/behavior embedded in the structure of the temporal data [2]. The time series analysis has significance in many domains, such as predicting the electricity demand, forecasting weather and stock prices, and learning about the behavior of usage analysis including electricity, fuels, etc. 

In this paper, we have studied a real-time data series to learn and identify a certain pattern indicating interesting behavior. Learning these behaviors can lead us to make smart decisions at the appropriate time to maximize the performance of a system deployed in a home or any other scenario. In the next subsection, we have explained the target scenario in detail. 

### Target Scenario

**Figure 1:**
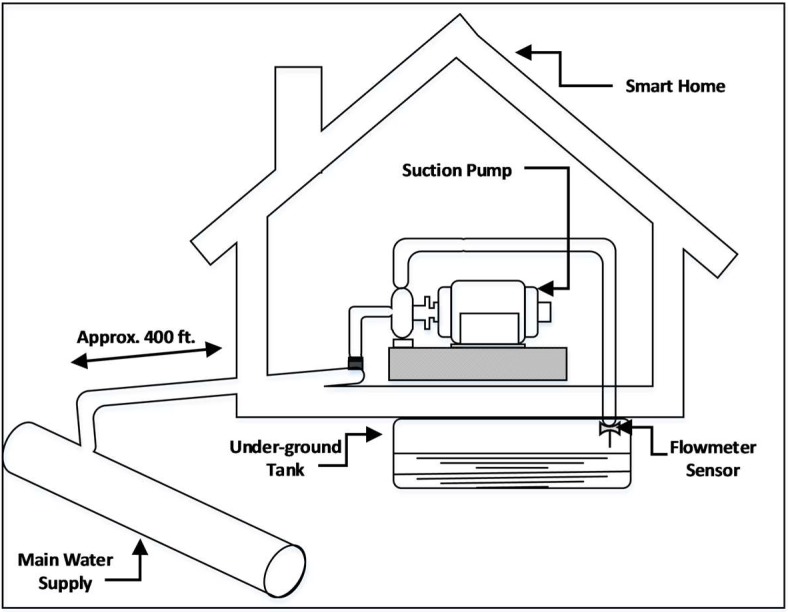
Depiction of key components of WMS.

The Water Management System (WMS) is one such system which monitors and controls the flow and reservoir of water in a home. This system primarily consists of a hydraulic suction pump that suctions water from an outside source into an underground storage tank. A sensor flowmeter is installed on a line which connects an outside water source with the underground tank. The flowmeter’s task is to measure the flow of water coming into the tank through the suction pump. Since the water availability from the outside water supply is highly unpredictable, a method is required to ensure that the pump turns ON at the appropriate time to suction the water when available. Moreover, the pump should not remain excessively ON as it could waste electricity, as well as air-lock the pump. The flowmeter senses the rate of water inflow at every minute which is then sent over the internet to the server using an Arduino board to create an online log file. The components relevant to this study are shown in Figure 1.

**Figure 2:**
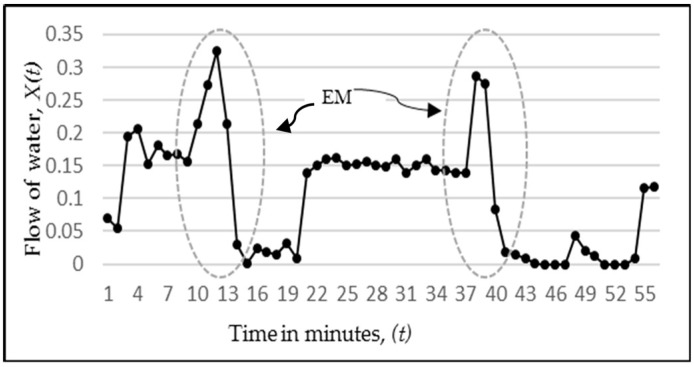
Normalized sample of flowmeter data having “Ending Marker” (EM)s.

The present study deals with the flowmeter sensor that detects the ending of water in the main water line. The inflow of water series will henceforth be considered as a discrete time series: X(1), X(2), …, X(t), where X(t) refers to the volume of inflow of water taken at discrete time intervals of one minute. This time series typically exhibits a peculiar behavior at the time when the outside source runs out of the water. The behavior happens just a few minutes before the water stops as the water level goes through a sudden spike and then it goes down to zero. The reason for this behavior can be that the water pipeline is drained out of the water and due to the air intake and the resulting reduction in back pressure, the water flow increases for few minutes, and then it falls to zero. For the sake of simplicity, we will refer to this behavior as the Ending-Marker (EM) in this paper and it is depicted in Figure 2. The behavior is observed many times in the series whenever the water is about to end in the main line. Our objective in this study is that if this behavior could be learned by a model, then the best time to turn OFF the suction pump can be predicted. This prediction is crucial not only to automate the whole system, but also to optimize the utilization ofresources (water storage, electricity).

While there are various algorithms and statistical techniques such as Auto Regressive (AR), Auto Regressive Moving Average (ARIMA), etc. to analyze temporal data [3], these statistical techniques work best when the data has a linear structure or can be converted to a linear structure. ANN are known for learning from imprecise and complex data which may contain interesting patterns and important trends. Learning these patterns and trends is a very complex task because it requires an analytic technique which is flexible enough to capture the dynamicity of the system. ANN is a technique which is not only adaptive, data-driven, and self-organizing, but also has the capability to learn the dynamic nature of the problem using real-time analysis [4]. The streaming data used in this study is the inflow of water measured by the flowmeter sensor. It has wide-ranging randomness in its magnitude, making predication a challenging task. It also has temporal variations and dependencies which need an algorithm that can adapt to temporal behaviors. Moreover, it is also non-linear in structure, making it impossible to use a well-known statistical technique to analyze temporal data. Hence, these aspects found in the sensor data under study have made the ANN algorithm an appropriate choice for the problem at hand.

In this study, three types of ANN are investigated for multi-step-ahead (MSA) forecasting and their performances are compared for predicting the underlying behavior in the flowmeter data. These models are explained briefly in the next section (Section 2.1). As a feasibility study, we have initially applied single-step-ahead forecasting on a synthetic dataset (Section 2.3) which we created and is of a similar nature as the flowmeter data. The synthetic data was used as a proof of concept to determine the applicability of the ANN model in learning behavioral patterns in the data stream. 

The rest of the paper is organized as follows. Section 2 presents the technical background of our study, a brief overview of other relevant work, and a preliminary analysis applied to synthetic data. Section 3 describes the approach and the model used in this study, while Section 4 presents the experiments, results, and discussion. Finally, Section 5 concludes the work by summarizing the main findings.

## 2. Background and Preliminary Analysis

### 2.1. Technical Background

The models studied in this work are Multi-Input Single-Output (MISO), Multi-Input Multi-Output (MIMO), and Serial Propagated/Recurrent Neural Networks (RNNs) [5]. All three models treat the temporal data as time series X(1), X(2), …, X(t), where the values till time *t* are used to predict X(t+1) to X(t+m), where *m* > 1. A brief introduction of these models is given below.

#### 2.1.1. MIMO

**Figure 3:**
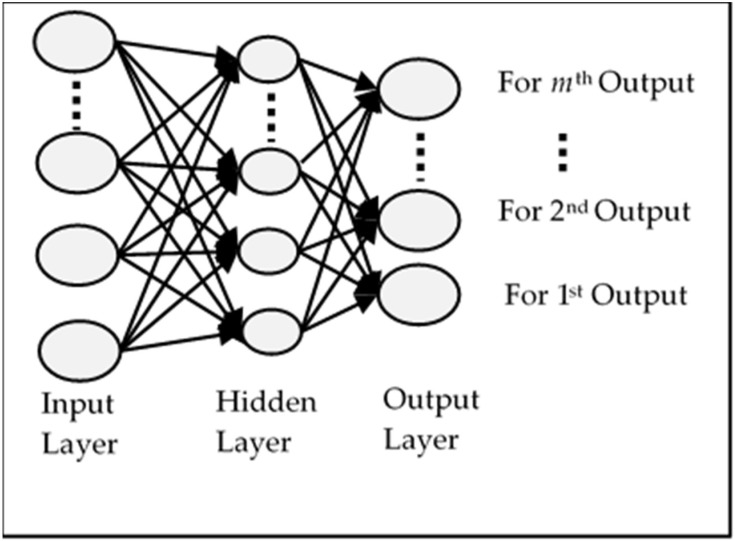
Structure of a typical MIMO Network.

For MSA, MIMO models are considered to be the most common and the simplest approach [6]. In this approach, a single neural network is created where the input layer contains the neurons equal to the size of the sliding window and the output layer contains the neurons equal to the step-ahead predicted values (*m*). A typical structure of MIMO is shown in Figure 3. Since a single network is created for all outputs, the weights are learned in a way to optimize a multi-objective function which optimizes all outputs. The training time is also larger than a single-step-ahead forecasting network because there is a larger number of weights.

#### 2.1.2. MISO

**Figure 4:**
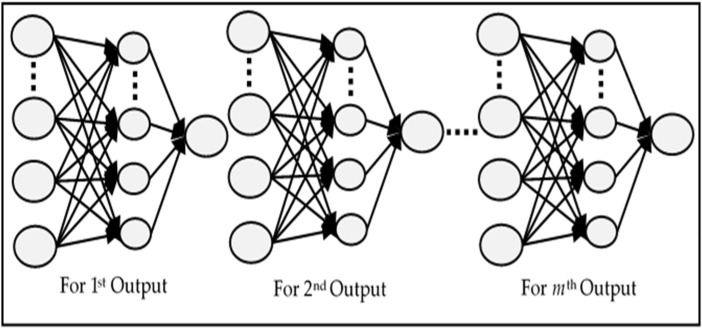
Structure of a typical MISO network.

In this type of ANN, multiple independent networks are created which are equal to the number of outputs. Each network is trained to produce a single output which makes such models useful for parallel computation [7], where the data volume is huge. A typical structure of the MISO network is shown in Figure 4. Since every network’s weights are optimized according to one output only, this not only increases the accuracy, but also reduces the learning time. Each individual network has a lower number of weights between the hidden and the output layer than the MIMO network. The collective time of training of all independent neural networks is much higher than that of MIMO. Another drawback is that the predictive performance of the network decreases as we increase the value of *m*.

#### 2.1.3. RNN

**Figure 5:**
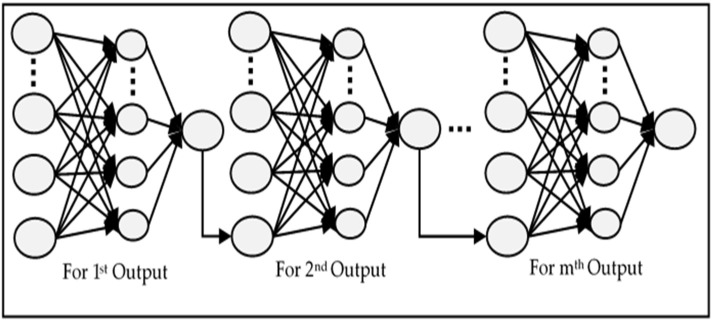
Structure of a typical RNN.

In RNN, also known as the serial propagated network, *m* multiple neural networks of identical structures are created where each of these networks is a single-step-ahead predictor to produce *m* predictions respectively. These networks are combined to form one single network in such a way that the output of the previous network is fed as the input to the next network. They are similar to MISO but have the advantage of using previous outputs in making future predictions. Figure 5 shows the structure of RNN. The feedback loops in an RNN make it possible to exhibit dynamic temporal behavior. The main concern of this model in MSA forecasting is that the noise or the error is also multiplied and propagated to successive predictions. If the data has lots of pattern variations and fluctuations, then this can lead to larger errors which multiply as the future steps are increased. This problem can be dealt with to a certain extent by using a proper threshold of errors at the output level to prevent them from propagating to the next prediction.

### 2.2. Literature Review

This section presents a brief literature review to explore the application of the various types of ANN in real-time forecasting. To analyze and forecast the temporal data, statistical methodologies like Autoregressive Integrated Moving Average (ARIMA) have been used to fit the class of linear time series models [8]. The restriction of linearity has been addressed by many researchers and, as a result, various variations of ARIMA models have been proposed [9]. However, each of the proposed models has its own set of limitations. ANN is another algorithm which has been studied as an alternate approach to model the non-linear relationships in data [10]. This technique is not only capable of learning the non-linear structures, but also of learning the underlying complex pattern from the data. In [11], besides researching the uses of ANN in non-linear models, the authors also highlighted the important developments in real-time data forecasting by ANN. There have been various studies where ANN is applied successfully to analyze the real-time data in domains such as electricity load forecasting [12,13,14], stock price prediction [15,16], commodity price prediction [17], etc., as explained further below.

In [12], the authors used the combination of clustering and the Bayesian Neural Network (BNN) for short term load forecasting of electricity. K-Means clustering was used to identify the most appropriate training data to forecast an hour-ahead value, whereas BNN was used as a forecasting model. The results obtained using BNN claimed to have the highest accuracy compared to several other models. Similarly, there is another study which forecasts hourly load data, using ANN on the basis of real-time data [13]. The researchers not only used the past load information, but also used the meteorological factors including humidity level, temperature, air pressure, wind speed, and visibility to predict the next hour load and the results showed improvement in terms of the performance. Electric load forecasting was also accomplished via Fuzzy Neural Networks (FNN) [18], where three different weight learning algorithms were used to learn the network. The study not only forecasts the peak-load and average-load, but also provides a high-level understanding of the neural network by converting an expert system into a Neural Network (NN). Whereas, in [14], the authors have studied the theoretical basis of the BP (back propagation) neural network and used it to conduct short-term power load forecasting.

ANN has also been used largely in stock price predictions. In [15], pattern recognition is studied on stocks temporal data and ANN has shown promising results in stock price prediction and pattern recognition. In another study [16], a combination of ANN and ARIMA is used to study the stock prices. Here, the authors not only predicted the price indices, but also made an effort to learn the behavioral pattern in temporal data. 

In [17], the price of crude palm oil (Soy bean oil) has been predicted using ANN, where the data was comprised of the historical price of Soy bean oil and the currency exchange rate. Here, the authors have studied the performance of the model by changing the network structure to get the best results. 

ANN has applications in many other areas of time series data prediction. A group of researchers proposed a design to use distributed computing power for training multiple time series and studied various machine learning algorithms for time series prediction [19]. They used Hidden Markov Models (HMM) for short term dependencies like temperature and the Recurrent Neural Network for long term dependencies like wind speeds. Neural networks have also been successfully used for analyzing the traffic time series [20] and predicting the traffic in one direction of the road segment. A feed forward neural network was used which takes the traffic data of the last few days to predict the next day traffic volume.

There are various studies where ANN has been successfully used for monitoring and controlling the resources in a smart home [21]. We have also used various ANN techniques to learn the behavior of streaming values of water inflows in a smart home. The novelty of our work is the application of ANN to learning the unknown patters that are often found in a sensor’s data and to use this in making a predictive system for automated decision making. In this methodology, we evaluated different models’ performance on the basis of the lowest MSE and then used this model to automate a decision process in a smart home. To the best of our knowledge, this is the first use of ANN for the purpose of analyzing the streaming data of water inflow to automate a water management system in a smart home or building.

### 2.3. Preliminary Analysis Using Synthetic Data

**Figure 6:**
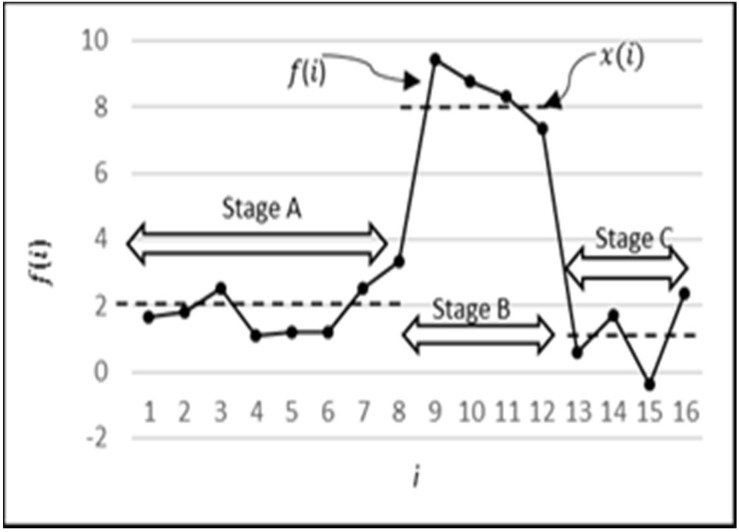
Details of the pattern created in synthetic data.

To determine whether ANN can be used for the purpose as specified in Section 1, we initially created a series of synthetic data f(i) for testing the applicability of ANN. The f(i) mimics, under controllable parameters, the known characteristics of the EM which are exhibited by X(t) and are created as follows. The dashed line in Figure 6, referred to as the baseline x(i), indicates the deterministic expected behavior of the EM. To make the EM randomized with controllable parameters, we have introduced random fluctuations y(i) in the EM. The baseline, along with one realization of the fluctuations, is shown as the solid line in Figure 6. The different stages of EM have been depicted as stage A, stage B, and stage C. Stage A consists of n data points where n is a uniformly distributed number between 1 and 30. Stage B and stage C consist of four data points each. The randomization is done by adding a uniformly distributed random value (y) ranging from −1.5 to +1.5 to the baseline. Hence, the equation is:

f(i)=x(i)+y(i)

$$x\left(i\right)=\;\left\{\begin{array}{c} \underline{Baseline\;Values}\\ 2\;-stage\;A\\ 8\;-stage\;B\\ 1-stage\;C \end{array}|\begin{array}{c} \underline{Length}\\ 1-30\;\left(n\right)\\ 4\\ 4 \end{array}\right\}$$

(1) y(i)=UDist. ∈[−1.5, +1.5]

**Figure 7:**
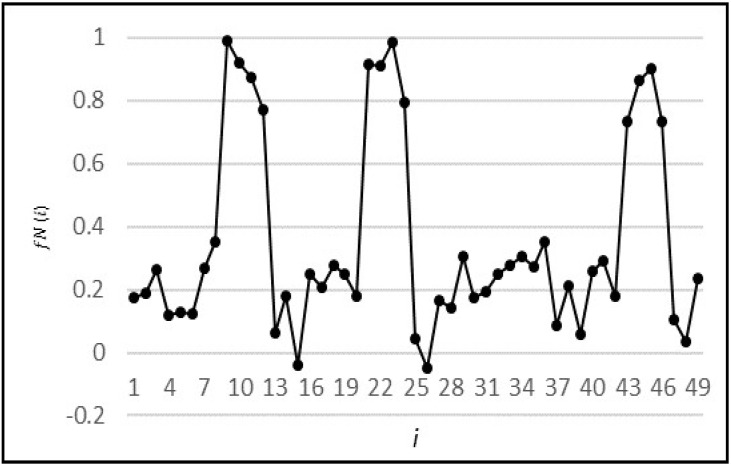
Example realization of normalized synthetic series.

The pattern of Figure 6 was repeatedly created with a series of non-repeating random values to generate 12,000 time intervals. Normalization of f(i) was done using Equation (2) to ensure that the data was in the range between 0 and 1. A sample series of normalized synthetic points $f^{N}\left(i\right)$ is shown in Figure 7.

(2) $$f^{N}\left(i\right)=\frac{f\left(i\right)}{M},\;where\;M=Max\;f\left(i\right)\;\forall i$$

The synthetic flow data is taken to be the time series $f^{N}\left(1\right)$,…,$\;f^{N}\left(i\right)$ format to predict the next value $\hat{f}\left(i+1\right)$, where previous *p* values of the data stream shall now be used. As given in Equation (3):

(3) $$\hat{f}\left(i+1\right)\;=h^{1,p}\left(f(\left(i\right),f\left(i-1\right),\;\ldots \;,f(i-p+1\right)),\;where\;p=\;number\;of\;previous\;values\;used$$

**Figure 8:**
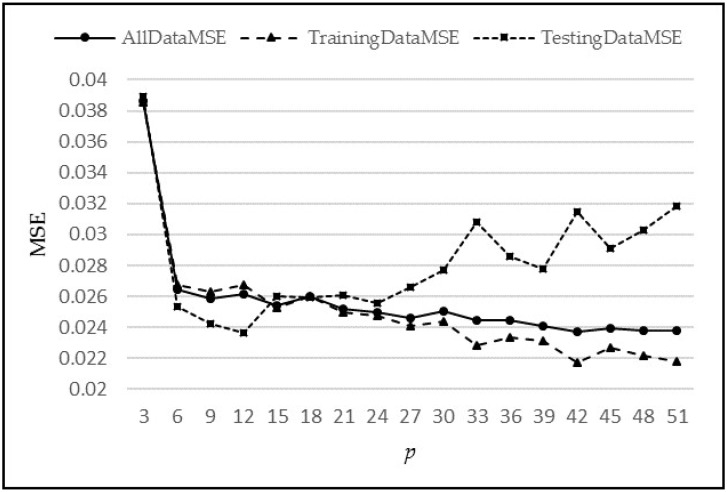
MSE vs. Lags for synthetic data.

The initial model $h^{1,p}()$ is the single-step ANN model used for single-step-ahead prediction. It is very simple in terms of its structure, having one hidden layer of five neurons, where the purpose is to test its predictive power on synthetic data. Multiple experiments were then performed with different values of p, varying from 3 to 51, to determine the most suitable value referred to as p*. The selection of p* was based on an analysis of Mean Squared Error (MSE), as plotted in Figure 8. The error is defined as the difference of actual flow and the predicted flow, i.e., $f^{N}\left(i\right)-\;\hat{f}\left(i+1\right)$. In this figure, it can be seen that the maximum drop in MSE is at *p* = 6 (MSE = 0.025). It should also be noted that the MSE does not decrease much beyond *p* = 6 and in fact, beyond 24, it starts to increase, most likely due to over-fitting. Since the complexity also increases with an increase in *p*, the most suitable value for *p* is chosen as p* = 6.

**Figure 9:**
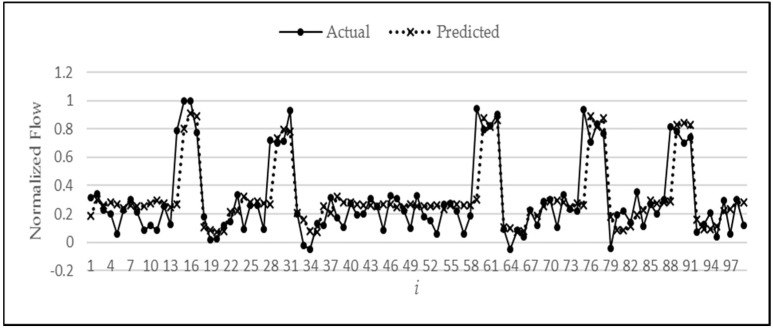
Plot of actual vs. predicted values of synthetic test data.

Next, the actual synthetic data values ($f^{N}\left(i\right)$) and predicted values $\hat{f}$(i), using a p* value of six, are analyzed to study the performance of the ANN model on the test data. Figure 9 contains the plot of actual versus predicted values for synthetic test data.

If we analyze the graph in Figure 9, we can see that the first point where the flow value reaches a sudden high value is always missed and cannot be predicted. This misprediction at this initial point is quite understandable since this sudden rise is a random behavior and could happen at any time in the series. But after this initial rise, the model is relatively accurate in predicting the remaining portion of the pattern. In particular, the model does not miss the point where the values suddenly drop to zero. This sudden drop is predicted quite accurately. Table 1 shows the MSE corresponding to each of the three stages. As expected, stage B can be seen to have the highest MSE, which is due to the understandable misprediction of the first point of sudden rise. Hence, accumulation of this magnitude of error has given the largest MSE to stage B predictions.

This experiment of ANN with a synthetic controlled data set has proven that an ANN can be used to efficiently learn and predict repeating behaviors in a data stream. To apply ANN to the actual flowmeter data series, we now carry out a more in-depth analysis of three types of ANN with complex structures and different parameters in the next section.

## 3. System Model

### 3.1. Overall System Description

Having now understood the effectiveness of using ANN, we now apply models on real-world flowmeter data. The actual flowmeter data contained a record entry for every minute, where each entry contained the date, time, and amount of inflow of water. For this analysis, only the rate of water inflow variable is used, denoted as g(t), with *t* being the integer values denoting the time variable in minutes. The data series, shown in Figure 2 and also discussed in Section 1, had shown a specific pattern that existed in the flowmeter data and was marked by the dashed circle labeled as EM. Our target is now to learn and predict this pattern and decide on its basis, the controlling of the suction pump (ON/OFF). To accomplish this task, we now go through a two-stage process. In the first stage, three types of ANN are applied to the flowmeter’s data for forecasting future flow and the best one is selected based on the MSE performance. In the second stage, a Decision Support System s(t) is constructed which decides either to keep the suction pump ON or turn it OFF based on *m*-step ahead predicted values. Since the output of ANN will be directing the decision, a single-step-ahead prediction is not sufficient to make this decision. To make an informed decision, an MSA prediction is needed which gives an *m*-step prediction of the future flow where m is now greater than 1. We refer to the *m*-step-ahead model using p previous values as $h^{m,p}\left(t\right)$. For this purpose, several experiments were separately conducted to determine the optimum value of *m*. A value of *m* less than three was not found to be sufficient to make a decision and a value more than three caused a larger error accumulation, leading to an incorrect decision (This can be further proved by Figure 15b,c where the two-step and three-step ahead predictions are shown, respectively). Hence, a value of *m* = 3 was found to be appropriate. Hence, the *m*-step-ahead prediction is given by Equation (4), where $g^{N}\left(t\right)$ is the normalized value of g(t), as in Equation (2).

(4) $$\hat{g}\left(t+r\right)=\;h^{r,p}\left(t\right)=\;h^{r,p}\left(g^{N}\left(t\right),g^{N}\left(t-1\right),\ldots ,g^{N}\left(t-p+1\right)\right),\;where\;r=1,2,\ldots ,\;m$$

To implement the second stage of the process, a threshold value of *l*(*t*), denoted as *T_f_*, needed to be selected because the decision model, *s*(t), is based on its value. The criteria used in *s*(t) were as follows: if all three predicted values are below $T_{f}$, then the decision is to turn the pump OFF, else, it is left ON for the next reading, as given in the equation below.

(5) $$s\left(t\right)=\{\begin{array}{cc} 1\;\left(pump\;ON\right), & if\;\hat{g}\left(t+1\right),\ldots ,\hat{g}\left(t+m\right)\;\forall >T_{f}\\ 0\;\left(pump\;OFF\right), & Otherwise \end{array}$$

**Figure 10:**
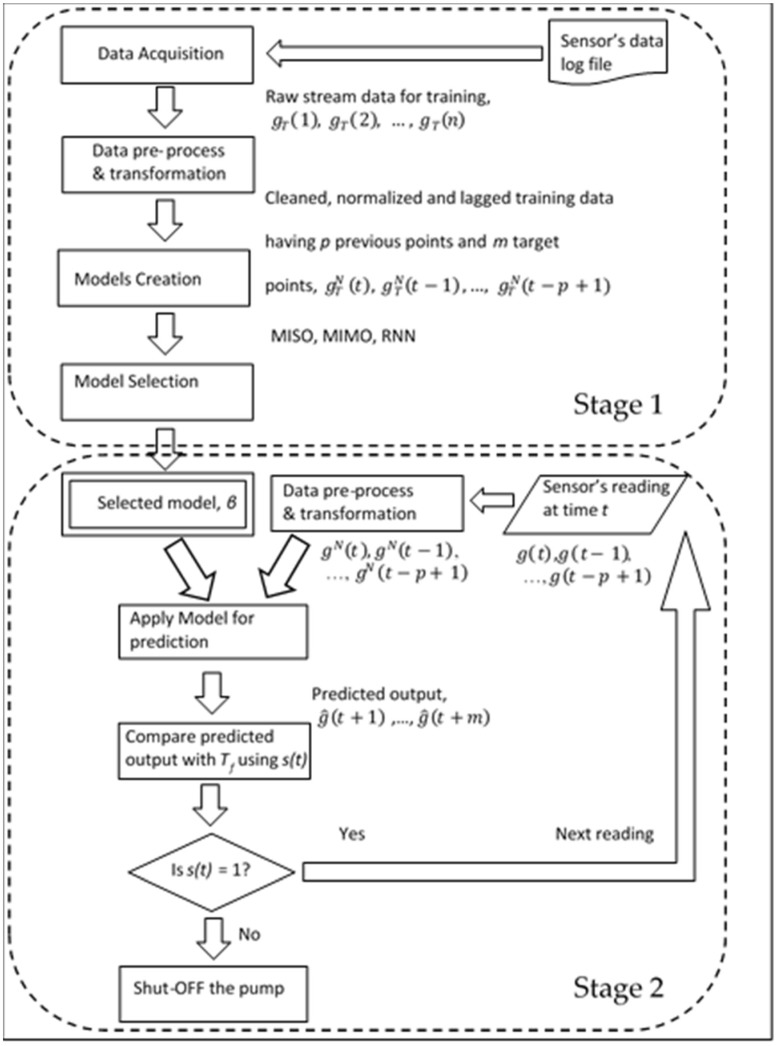
Complete process diagram comprising two stages.

At every reading, the predicted values are compared against a threshold value $T_{f}$ and the decision is made until the pump shuts OFF. The complete cycle till the pump is turned OFF is shown in Figure 10. Here, we represent $g_{T}\left(t\right)$ as training data.

### 3.2. ANN Model

As a first step, the data was preprocessed by dealing with type errors and missing values and it was normalized using Equation (2), as mentioned in Section 2.3. Later, it was converted in the form of time series for different values of *p* (lags), where *p* = number of previous values. As done for the synthetic data, the flowmeter series was converted into many lagged datasets using *p* from 2 till 12 and the most suitable *p* was selected by plotting MSE against the number of previous values (*p*) graph. The structure of the ANN model, for all types (MIMO, MISO, RNN), contained an input layer having neurons equal to the number of lags (*p*) in the dataset. The range [2,12] is selected because the EM pattern, which we are trying to detect, comprises four to five data points at its maximum. In addition, increasing the neurons at the input layer increases the network complexity, which effects the overall performance. The output layer contains neurons equal to *m*, the MSA predictions, which in this study is set to be three for the reasons discussed above. As far as the hidden layers and hidden neurons are concerned, there are two possible ANN architectures. The first model is the one which uses a single hidden layer, while the other is the one in which multiple hidden layers are used. It has been shown in the literature that a single hidden layer with a larger number of neurons reduces complexity and hence is preferred to the more complex multi hidden layered model [22]. Hence, the former approach is used in our paper. The number of hidden neurons (*N_h_*) is selected by the rule of thumb, which states that the number of hidden neurons should be in the range of the number of output neurons (*N_o_*) and the number of input neurons (*N_i_*) [22]. This was further confirmed in separate experiments when the model was over-fitted with a higher number of hidden neurons and under-fitted with a lesser number. Hence, we can calculate the number of hidden neurons according to Equation (6). The number of hidden neurons calculated for each value of *p* is given in Table 2.

(6) $$N_{h}=\lceil \frac{\left(N_{i}+\;N_{o}\right)}{2}\rceil$$

**Figure 11:**
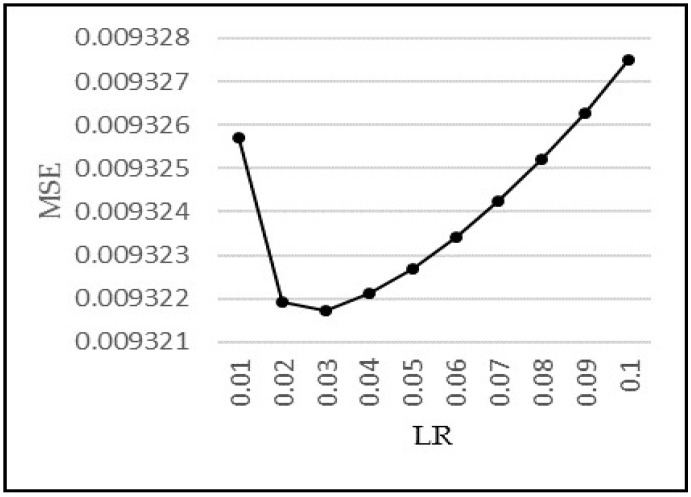
MSE vs. Learning Rate.

The ANN models require the determination of Learning Rate (LR), which should be low enough so that the model converges to something useful and high enough so that the training time can be minimized. To determine the optimum value of LR, we kept the other parameters of our model constant and used multiple values of LR to train the model and obtain corresponding MSE. We then plot the MSE against corresponding LR, as shown in Figure 11. The optimal value of LR can then be found by the graph where MSE is minimum and is shown to be 0.03, which is henceforth used in our models.

The Back-propagation algorithm with gradient descent is used for learning weights while the Sigmoid function is used for activation as they are the most commonly used techniques in ANN [22,23]. For training the ANN, K-fold cross validation is used since it has been suggested in the literature that K-fold is an ideal choice for small datasets [24] Here, we have used K-fold with a value of k = 5. 

**Figure 12:**
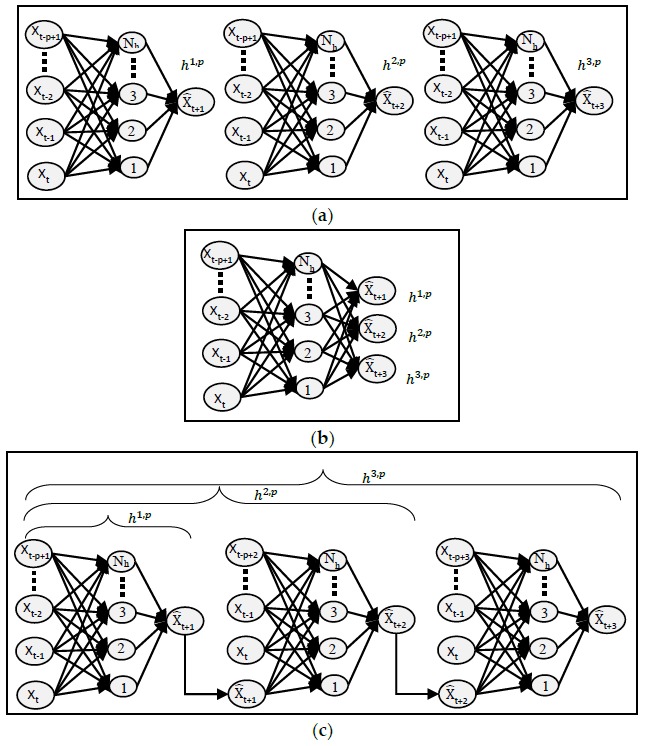
(**a**) MISO for three outputs; (**b**) MIMO with three outputs; (**c**) RNN model (unrolled) for three outputs.

Three models of ANN, that is, MISO, MIMO, RNN, as described earlier in Section 2.1, are tested and are shown in Figure 12a–c, respectively.

## 4. Experiments, Results, and Discussion

**Figure 13:**
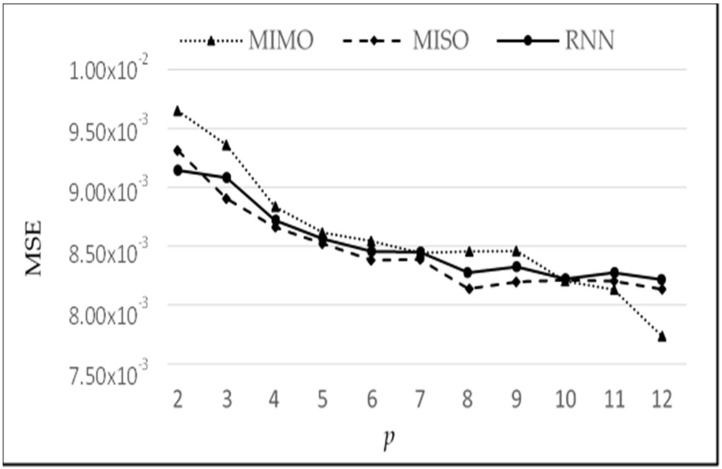
MSE vs. Lags for MIMO, MISO, and RNN.

We now determine the most suitable value of *p* based on the graph of MSE plotted against each lag *p* and by the analysis of percentage decrease in MSE as *p* increases. Figure 13 shows the graph of MSE vs. *p*. A suitable choice of *p* (p*) value is based on a trade-off between reducing MSE vs. increasing the complexity of the network. As, we increase *p*, the MSE decreases; however, the complexity also increases. A larger value of *p* requires larger numbers of neurons at the input layer, which in turn requires a larger number of weights in the neural network. The weights are learned in an iterative manner, so the larger the number of weights needed, the more time and space is required for convergence, hence, increasing the time and resource complexity of the system. 

Table 3 shows the relative reduction in MSE as *p* is increased on a step by step basis. It may be noted that as *p* is increased, the percentage decrease in MSE at each stage keeps on reducing. Since each increase in p also increases the complexity of the system, we heuristically chose *p* = 5, where the reduction in error is 1.98%, while the subsequent increase in *p* will only decrease the percentage error by 1.2%, as shown in the last column of Table 3.

**Figure 14:**
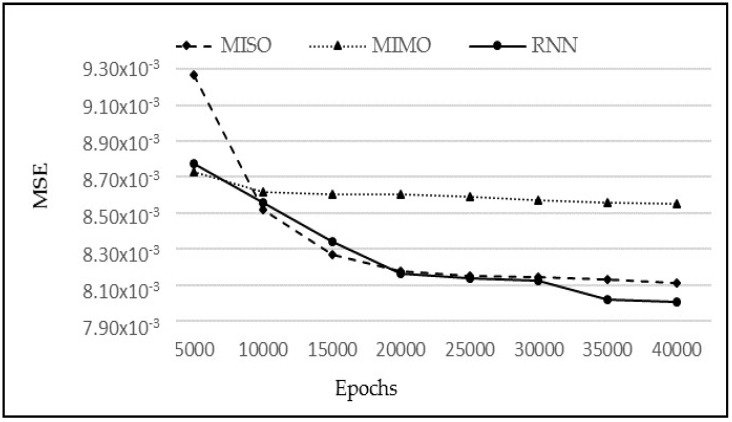
MSE vs. Epochs for MISO, MIMO, and RNN.

The comparative analysis of models MIMO, MISO, and RNN can also be seen in Figure 13. MIMO generally performed the worst as it has the largest MSE for each value of *p*; however, it is not very different from the other two models. Similarly, MISO and RNN have very close results, but MISO generally outperformed RNN. To analyse the performance of these models more closely and deeply, these experiments have been run again with varying learning iterations. Initially, 10,000 epochs were used to train the model. The models were then tested between 5000 to 40,000 epochs to see the effect of a longer training time on every model. The other parameters were kept the same while *p* = 5 was used for the input layer. The results are shown in Figure 14.

**Figure 15:**
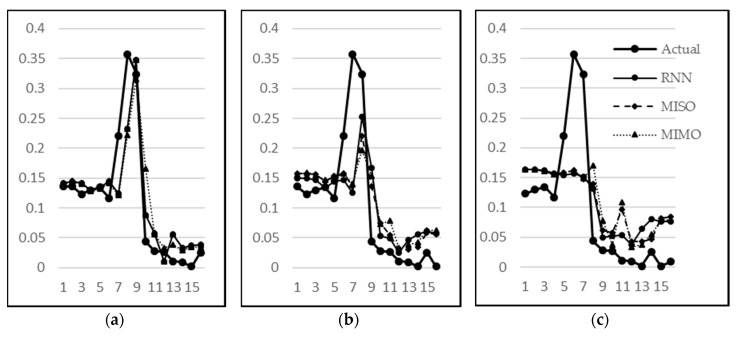
Actual vs. Predicted plot for (**a**) one-step-ahead forecasting; (**b**) two-step-ahead forecasting; and (**c**) three-step-ahead forecasting.

From Figure 14, it is clear that there is almost a constant performance by MIMO after 10,000 epochs; showing no change in the MSE as the training time progresses. As far as the MISO and RNN are concerned, both show a very similar performance between 10,000 and 30,000 epochs. However, after 30,000 epochs, there is a slight drop in MSE for RNN, giving the notion that if the algorithms are trained for a longer period of time, then RNN may slightly outperform the other models for this dataset. However, the difference above may not be statistically significant and all three techniques continue to be subsequently analysed in the remaining experiments. Next, their respective observations and predictions for one-step–ahead, two-step-ahead, and three-step-ahead are analysed in Figure 15a–c, respectively. The sudden rise (stage B) in water level is less and less predictable in one, two, and three step-ahead predictions, respectively. However, the subsequent behaviour (stage C) is predictable with a reasonable accuracy even in three-step-ahead predictions. It can also be seen that MISO and RNN have almost similar predictions for one-step-ahead predictions, which is intuitively correct since there is no difference in both models for this category. Their performance, however, differs for two-step-ahead and three-step-ahead predictions since RNN utilizes the previous predictions for the next prediction. Hence, overall RNN has performed better than MISO and MIMO when considering the collective performance of all three outputs. 

**Figure 16:**
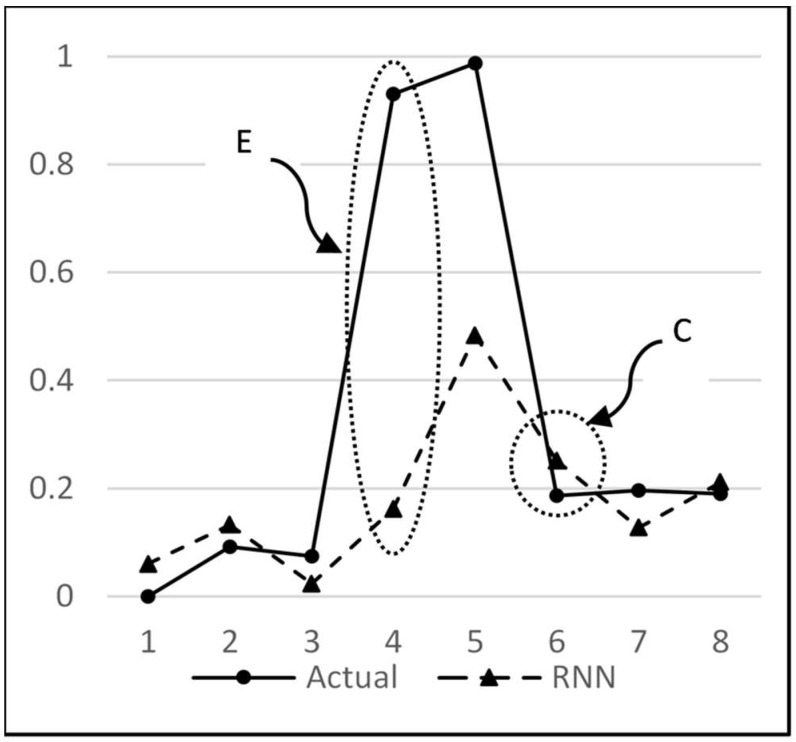
Points of misprediction marked by E and points of correct prediction marked by C.

An analysis of the predictions by RNN and MISO for all possible combinations reveals that both of these models have a similar performance. Ideally, a large difference in performance was expected with RNN because of the advantage that it uses the previous outputs to make the next predictions. But a close analysis has revealed that the same advantage is the reason for not displaying a remarkable difference when compared to MISO. This can be explained by the graph in Figure 16. Here, the magnitude indicated by circle E is the error amount in predicting the sudden rise in water flow, which in turn is propagated back to the network as an input to determine the next output. This accumulation of error in multi-step-ahead predictions lowers the anticipated performance of the model. This sudden rise in water flow is an unpredictable behaviour since its occurrence is random and can happen at any point in time. What is predictable, however, is that after going through a sudden rise, the water stays there for one or two readings, and then it goes down to zero. Hence, this explains why even though RNN is the overall best performer, it does not outperform MISO to the extent expected. It may be noted that the above behaviour has been partly subsequently predicted by the RNN model, as marked by the circle C in Figure 16. At point *t* = 5, the actual flow is 0.96, and at point *t* = 6, it is 0.2. The prediction by ANN at point 5 is 0.5, but at point 6, it predicted that the flow would not continue to stay high and would fall to zero, regardless of the fact that it is high in the most recent readings. Hence, it was correct in learning the pattern and making a prediction close to the actual value of 0.2.

Once the models are learned, they can potentially be used as a decision support system to decide the state of the suction pump. The system would take *p* = 5 five most recent readings of the flowmeter sensor, apply RNN to it, predict *m* = 3 future outputs, and suggest a state of the pump. If all three predicted outputs are below the threshold (*T_f_*) value, then the pump would be turned OFF; otherwise, it would be kept ON until the next reading. The same procedure will be repeated for every reading until the pump is turned OFF by the system or by an external source. To evaluate the performance of this system, 100 cases from the flowmeter data series are extracted. The series exhibit four distinct types of behaviours, labelled as categories, which are listed in Table 4. The count of each case is also shown. Please note that the count of each category does not necessarily reflect the frequency of occurrence of each category and this factor is not relevant to the result.

All the three models, along with their ensemble (a combination of MIMO, MISO, and RNN), were tested to find the model which gives the best accuracy along with other performance measures. The ensemble model is a committee model which uses the voting mechanism for prediction [25,26,27]. If the majority votes for the ON state, it predicts ON and if the majority votes for the OFF state, it predicts OFF. As explained earlier, the values predicted by these models are compared against a threshold value *T_f_* which needs to be determined. The most appropriate value of (*T_f_*) was based on a combination of two factors: (i) a judgment of what is an appropriate level of flow; and (ii) determination of the accuracy of the decision support system for which several values were tested, including 0.01, 0.025, 0.05, 0.075, and 0.1. For each value of *T_f_*, the corresponding accuracies are given in Table 5. After an analysis of these two factors, it was found that the most appropriate value of *T_f_* was 0.075, which further can be confirmed by Table 5, as it gives the maximum accuracy. Next, the performances of all the models using *T_f_* = 0.075 are given in Table 6.

Finally, the models were evaluated using the usual performance measures such as accuracy, precision, recall, and F-measure. Their equations are given below, where TP = true positive, TN = true negative, FP = false positive, and FN = false negative.

$$Accuracy=\;\frac{\left(\mathrm{TP}+\mathrm{TN}\right)}{\left(\mathrm{TP}+\mathrm{FN}+\mathrm{FP}+\mathrm{TN}\right)}$$

$$Precision=\;\frac{\mathrm{TP}}{\left(\mathrm{TP}+\mathrm{FP}\right)}$$

$$Recall=\;\frac{\mathrm{TP}}{\left(\mathrm{TP}+\mathrm{FN}\right)}$$

$$F-measure=\;\frac{2\mathrm{TP}}{\left(2\mathrm{TP}+\mathrm{FP}+\mathrm{FN}\right)}$$

**Figure 17:**
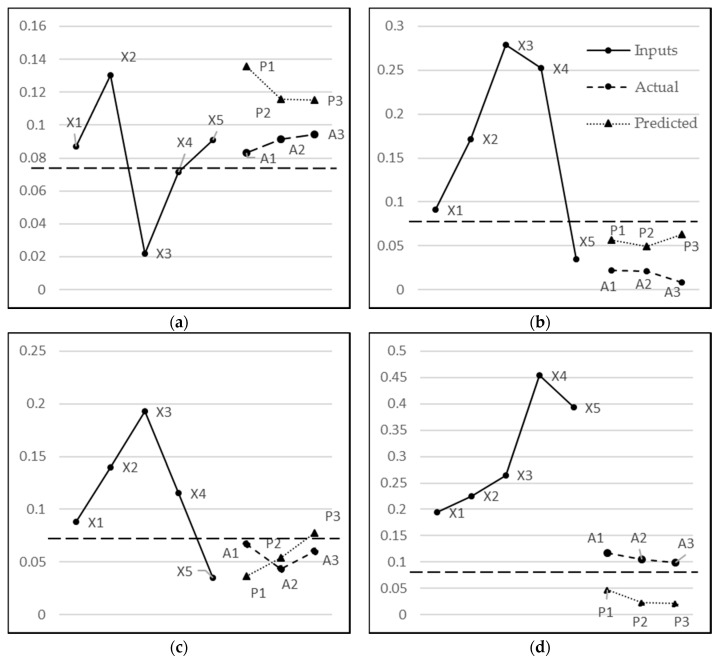
A representative case of each (**a**) TP (Actual & predicted decisions are ON); (**b**) TN (Actual & predicted decisions are OFF); (**c**) FP (Actual decision is OFF & predicted decision is ON); and (**d**) (Actual decision is ON & predicted decision is OFF) FN are shown where X1, X2, X3, X4, and X5 are the inputs; A1, A2, A3 are actual values; and P1, P2, P3 are the three-step-ahead predicted outputs. The dotted straight line at 0.075 is the *T_f_* for decision.

We have used the ON state of the pump as the positive class, while the OFF state is the negative class. In this study, FP is more critical than the FN measure because it represents those situations where the decision was taken to keep the pump ON whereas it should have been turned OFF. Similarly, TN is more critical than TP as it represents those situations where the pump should have been turned OFF and the same is suggested by the model. As shown in Table 6, RNN has outperformed all other models including the ensemble model in all of the performance measures. It gives 86% accuracy, 91% precision, 74% recall, and 82% F-measure values. A case representative of each, TP, TN, FP, and FN, is shown in Figure 17. For example, Figure 17b shows the TN scenario where both the actual and predicted values fall below the threshold and hence, the prediction is accurate. On the other hand, Figure 17d depicts the scenario where predicted values fall below the threshold and the actual values, while being quite low, are above the threshold. Hence, this falls under the case of FN predictions.

We now summarize the performance of RNN for each category in Table 7. Category 1 contains the cases having the peculiar behaviour which were referred to as EM. The model was applied to 49 such cases where the model has achieved more than 97% accuracy, indicating that the model is able to locate the correct time to switch off the pump in 97% of the cases. Similarly, the model has a good accuracy for Categories 3 and 4, exhibiting 95.65% and 70% accuracy, respectively. However, it has a low accuracy of 55.56% for Category 2 cases, where the pattern is observed but the water flow does not stop. However, the low accuracy in this category is not of significance since the frequency of occurrence of category 2 cases is quite rare (as described earlier for Table 4). In such cases, the ideal decision should have been to keep the pump ON, which our model misses more than 40% of the time. This is also understandable, since the sample that we extracted from the flowmeter data to train the model is mainly comprised of Category 1 cases. This was due to the reason that we were primarily interested in predicting the best time to shut off the pump. Hence, based on the above analysis, it can be seen that the RNN model has exhibited a good performance by multiple performance metrics to show its power of capturing the EM and making a successful automatic system based on its prediction.

In retrospect, it may be noted that there could be a simpler technique to shut-off the hydraulic suction pump using a fixed threshold time value, which shuts off the pump if there is no water flow below a threshold value for a certain period of time. However, using such a simple technique would clearly not be optimal and would result in an excessive ON time for the pump, thus wasting energy and degrading the performance of the system.

## 5. Conclusions

IoT enhances the quality of life by connecting the digital world to the real world via utilizing various sensors in everyday objects. These sensors sense streams of data, which in turn may contain behaviors of special interest (i.e., the EM behavior in this study). It may be noted that no assumption has been made in our model that requires previous knowledge of the pattern to train our proposed model using the Artificial Neural Network (ANN). The raw data from the sensors after necessary pre-processing was fed into our model. We have not explicitly used any parameters pertaining to the EM pattern in learning the parameters of our network. Thus, our model was capable of learning the patterns and predicting the future with specific prior knowledge of a specific pattern. However, the occurrence of a pattern which was learnable by our system allowed the system to have a better accuracy in predicting the future. This, in turn, helped in making an autonomous system to manage resources in a smart home/building.

While the specific parameters that have been used in the system have to a certain extent been both data-driven and heuristically determined, we believe that these parameters will continue to provide accurate predictions for the life of the system. The basis of our belief is drawn from the fact that the period from which the data has been selected covers a very wide period of about twelve months. However, the general applicability of this model to be used as an autonomous system is intended to be further explored in the future by expanding the dataset to cover subsequent independent epochs. This is expected to further confirm the model’s general applicability. It is suggested that these explorations to better incorporate the changes in the behavior of the system are updated in the model after a certain period (e.g., annually) to update the learning process. This process, if introduced, will require fine-tuning of the model on an occasional basis by incorporating any changes in the behavior of the data over a longer period of time.

This research found the Artificial Neural Network (ANN) to be useful in recognizing such behavior in real-time data. Three models of ANN, namely MIMO, MISO, and RNN, were applied for three-step-ahead predictions of a flowmeter’s data series. Among these models, RNN was found to have the best performance. It was then used to make an automated decision support system to decide upon the appropriate state of the suction pump. The model was evaluated using 100 cases of different categories, where it was found to provide 86% accuracy, suggesting that a decision based on such a model can make the correct decisions 86% of the time. 

In the future, this work may be extended to extract rules from neural networks as these rules can give further insight into the behavior. 

## Figures and Tables

**Table 1:** MSE corresponding to each stage.

Stages	MSE
Stage A	0.010358
Stage B	0.102454
Stage C	0.009227

**Table 2:** Number of hidden neurons used with respect to every Lags/inputs of ANN.

*p* (Lags/Number of Inputs)	*N_h_* (Number of Hidden Neurons)
2	3
3	3
4	4
5	4
6	5
7	5
8	6
9	6
10	7
11	7
12	8

**Table 3:** MSE of every model for corresponding *p* and change in MSE as p increase.

*p*	MSE(MISO)	MSE(MIMO)	MSE(RNN)	Average MSE	Change in Percentage
2	0.009312	0.009648	0.009143	0.009368	---
3	0.008903	0.009359	0.009082	0.009114	2.71%
4	0.008661	0.008836	0.008719	0.008739	4.12%
5	0.008521	0.008615	0.008561	0.008566	**1.98%**
6	0.008381	0.008543	0.008456	0.00846	1.23%
7	0.008387	0.008443	0.008449	0.008426	0.39%
8	0.008138	0.008456	0.008274	0.008289	1.6%
9	0.008196	0.008458	0.008324	0.008326	−0.44%
10	0.008211	0.008202	0.008222	0.008212	1.37%
11	0.008202	0.008131	0.008273	0.008202	0.12%
12	0.008133	0.007735	0.008216	0.008028	2.12%

**Table 4:** Description and details of categories in cases of flowmeter data.

Categories	Description	Count	Correct Decision
**Category 1**	The cases where the EM was observed and water flow stopped. (The correct decision should be to shut off the pump).	49	OFF
**Category 2**	The cases where the data had a similar type of EM but the water flow did not stop. (The correct decision should be to keep the pump ON).	18	ON
**Category 3**	The cases where the EM was not observed at all and water flow had random fluctuations. (The correct decision should be to keep the pump ON).	23	ON
**Category 4**	The cases where the EM was not observed but water flow stopped anyway. (The correct decision should be to shut off the pump).	10	OFF
**Total**		100	

**Table 5:** Accuracies corresponding to each *T_f_*.

*T_f_*	Accuracy
0.01	42.00%
0.025	42.00%
0.05	81.00%
**0.075**	**86.00%**
0.1	82.00%

**Table 6:** Performance measures by all the models where *T_f_* is kept at 0.075.

Models	Accuracy	Precision	Recall	F-Measure
MIMO	82%	85.30%	69.05%	76.32%
MISO	82%	83.33%	71.43%	76.92%
**RNN**	**86%**	**91.20%**	**73.81%**	**81.58%**
Ensemble	84%	88.24%	71.43%	79%
**Best**	86%(RNN)	91%(RNN)	74%(RNN)	82%(RNN)

**Table 7:** Details of decisions made by RNN for each category.

Category	No. of Cases	No. of Correct Decisions	No. of Incorrect Decisions	Percentage of Correct Decision
1	49	48	1	97.95%
2	18	10	8	55.56%
3	23	21	2	91.67%
4	10	7	3	70%
**Total**	**100**	**86**	**14**	
**Percentage**		**86%**	**14%**	
